# Preferences of ICU Nurses for Improving Their Work System: A Sequential Exploratory Mixed‐Methods Study

**DOI:** 10.1111/nicc.70350

**Published:** 2026-02-02

**Authors:** Atefeh Mohammadinejad, Peyman Piranveysey, Rosanna Cousins, Hamidreza Mokarami

**Affiliations:** ^1^ Tarbiat Modares University Tehran Iran; ^2^ Shiraz University of Medical Sciences Shiraz Iran; ^3^ Liverpool Hope University Liverpool UK

## Abstract

**Background:**

Patients admitted to intensive care units (ICU) generally require expensive, advanced technological interventions as well as a high level of specialised and timely care to survive their medical crisis. Work in this setting can generate significant pressures for ICU nurses.

**Aim:**

To understand and prioritise ICU nurses' preferences for improving their work system based on the five components of the Systems Engineering Initiative for Patient Safety (SEIPS) model.

**Study Design:**

Using a sequential exploratory mixed‐methods design, qualitative interview data were first collected from ICU nurses employed in a tertiary hospital in Iran to identify interventions for improving their work system. Data saturation guided sample size. Directed content analysis was used for extracting themes representing areas for intervention. The qualitative data were then integrated into an Analytic Hierarchy Process for weighting and prioritising the identified interventions using quantitative pairwise comparisons and mathematical calculations.

**Results:**

Fourteen ICU nurses were interviewed. Directed content analysis of the transcribed data yielded 17 themes impacting nurses' well‐being and performance. The Analytic Hierarchy Process indicated that the themes with the highest priority for improving the ICU work system for nurses were Workload and Staffing, and micro‐level Support and Resources—associated with SEIPS Organisation and Person components, respectively. Notable intervention importance was also found for themes Collaboration and Support, Equipment and Technology Enhancement, Culture and Management, and Spatial Design and Arrangement.

**Conclusions:**

ICU nurses identified a high number of challenges in their work environment. Modern decision‐making analyses were able to quantify and prioritise their preferences. These provide direction for designing future interventions.

**Relevance to Clinical Practice:**

As technological advances continue to support patient survival, there is a need for judicious decision‐making in providing immediate and long‐term solutions for associated changes in the work system faced by critical care nurses to ensure nurse well‐being and patient care.

## Impact Statements

- What is Known About This Topic
  ○ The ICU work system is inherently stressful because it involves work with critically ill patients and life‐or‐death outcomes.
  ○ The ICU work system is dynamic and constantly evolving in terms of technical complexity, which indicates regular review of operational processes and timely intervention.
  ○ Critical care nursing has high turnover, which impacts both patient care and hospital budgets. There is a good business case for intervening to remove barriers in the work of ICU nurses.
- What This Paper Adds
  ○ Use of a robust mixed‐methods approach to identify 17 interventions across the ICU work system that warrant improvement according to ICU nurses.
  ○ First use of the Analytic Hierarchy Process method for multi‐criteria decision‐making to prioritise the identified interventions to progress an improved work system in ICU.
  ○ An approach and evidence‐based recommendations from ICU nurses to improve the work system, which can tackle high ICU nurse turnover and, in turn, improve patient care.
  ○ The work system of critical care nurses directly contributes to applying their expertise in monitoring and managing complex conditions towards patient survival and effective recovery. This article recognises that in pursuit of excellence, ICU nurses have raised issues about their work system, and working conditions are an important factor in the high turnover rates. This research sought to identify from experienced ICU nurses interventions that can improve the work system and how they can be prioritised for best effect. Application of the methodology and findings in ICU, wherever situated, can serve to support critical care nurses, and importantly, patient outcomes.

## 1 Introduction

The standard intensive care unit (ICU) environment is a specialised and advanced care setting to promote patient survival of medical crises. ICU are highly charged settings which often generate significant economic and psychological pressures due to the high stakes associated with care delivery [1]. As a result, ICU nurses face numerous challenges including high workloads [2], alongside shift‐working [3], which ultimately impacts on patient care [4]. Organisational factors, including insufficient support from superiors and role conflicts, can also affect nurses' ability to provide the necessary care [5, 6, 7]. Indeed, it has been reported that there are significant issues in all areas of the ICU when it comes to identifying performance obstacles [8, 9, 10, 11]. Understanding these multifaceted challenges is crucial for healthcare authorities to implement appropriate measures and support ICU nurses effectively [11]. Since many of the studies that have indicated barriers for effective nursing in ICU are pre‐pandemic, and the COVID‐19 pandemic changed much of the structure of intensive care [12, 13], it is important to have an up‐to‐date investigation of the ICU work environment for nurses. Additionally, there are now improved decision‐making models, which can support prioritisation of any need for intervention identified by ICU nurses to improve their own well‐being and performance and ultimately patient care. Thus, this article describes research that focuses on understanding what can be improved in the work system for ICU nurses, and from this, on understanding what should be prioritised when setting an agenda for intervention.

## 2 Background

As technology advances at a rapid pace and associated healthcare requirements continue to rise, budgets have generally struggled to keep up. In this context of limited resources and competing demands, policymakers are confronted with the important task of allocating public funds in an effective manner. This involves appropriate prioritising of system improvements and determining the right interventions that can be demonstrably effective. The challenge is that the decision‐making process regarding health interventions is complex and multifaceted [14]. Involving employees in intervention development and implementation can facilitate successful changes and intervention outcomes, including commitment to change and increased uptake of intervention activities [15, 16, 17, 18]. Certainly, utilising the expertise and contextual knowledge of ICU nurses within the organisation supplements the knowledge of intervention experts [19]. The process of improving the work system for healthcare workers should include attention to their well‐being [20]. Previous studies have shown that employees who perceived their experience has contributed to organisational‐level interventions and associated improvements in their work situation led to positive psychosocial outcomes [21, 22].

The literature includes some qualitative research on work system strategies from employees' viewpoints [23, 24, 25, 26]. This includes two studies of fatigue in registered nurses. In the first, interview data were used to provide recommendations to help nurses cope with work‐related fatigue, including teamwork, communication and mutual respect [25]. The second larger study by the same authors introduced the Systems Engineering Initiative for Patient Safety (SEIPS) model [27] to examine factors affecting fatigue in registered nurses in various departments, and the barriers and facilitators to nurse coping in hospital settings [26], as well as generally improving healthcare processes [28, 29].

**FIGURE 1:**
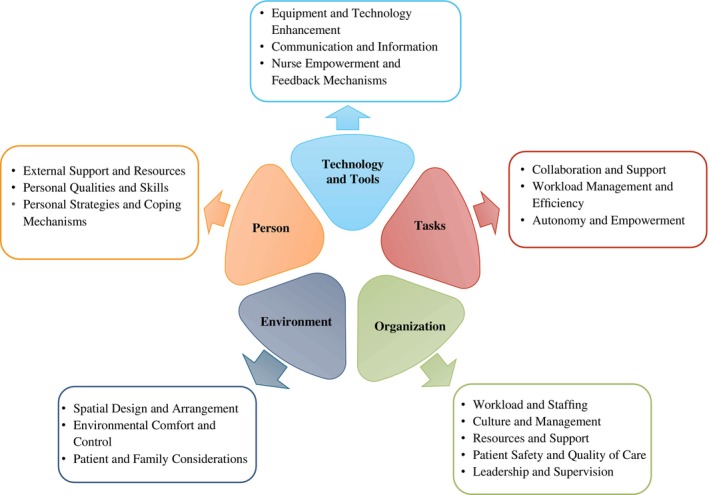
Visual of the five components of the SEIPS work system. (After Carayon et al., 2006 [27]).

The SEIPS model [27] was designed to enhance healthcare quality and patient safety by illustrating how work system components interact to influence patient safety (see Figure 1). It is widely used to guide healthcare quality improvement initiatives, showing how care processes and outcomes are shaped by work system factors, creating a feedback loop within the system [27, 30, 31]. In the SEIPS model, the Person (the nurse in this study) is central to the system, and the Person interacts with four other work system components, specified as Technology and Tools, Tasks, Environment and Organisation, all of which influence outcomes for patients, nurses and organisations [27].

Although there is evidence that work system characteristics impact upon nurses' workload, and suggestions that redesigning ICU work systems can enhance care quality and patient safety [9, 10, 31], it has been noted that the effectiveness of interventions varies based on contextual factors within work systems [32]. Moreover, further research is needed to develop a prioritisation system for work system facilitators and strategies using the SEIPS model.

## 3 Aim and Objectives

The aim of this study was to identify, quantify and prioritise work system interventions to improve ICU nurses' working conditions and well‐being, using the SEIPS model as the guiding framework. The first objective was to explore ICU nurses' perspectives on what aspects of their work system could be improved to enhance their performance in terms of patient care and safety and to reduce stressors affecting their well‐being, based on the SEIPS model. The second objective was to integrate the obtained data into an analytic hierarchy process (AHP) to generate recommendations for improving the ICU work system for the benefit of both nurses and patients.

## 4 Design and Methods

### 4.1 Design

The study used a two‐phase sequential exploratory mixed‐methods design. In the first phase, qualitative data were collected using interviews with ICU nurses for the purpose of identifying work system interventions that would be beneficial to ICU nurses' well‐being and performance in terms of patient care. The interview data were subjected to a deductive Directed Content Analysis [33] to identify themes and components based on the SEIPS model. Then, in the second phase, AHP was used to transform the themes identified in the qualitative phase into quantitative data through the ICU nurse participants' ratings of importance. The output of this assessment was then used to weight and rank the themes to drive forward the future development of interventions to improve the ICU work system for both nurses and patient care.

### 4.2 Setting and Sample

ICU nurses who provided direct patient care were recruited from a single tertiary care hospital in Fars, Iran. Critical care nursing is highly specialised, and additional qualifications are recommended [34], nevertheless, it is the case that some newly qualified nurses elect to specialise in ICU following their 3 years of general nurse training. To ensure that data were collected only from nurses fully integrated into ICU nursing, and beyond the initial period of gaining ward experience, and necessary time management, decision‐making and socialisation skills that may take 6 months [35], we applied an inclusion criterion of having at least 1 year of ICU experience, as well as personal willingness to participate in the study. A purposive sampling strategy was used to ensure a comprehensive understanding of the workplace dynamics affecting well‐being. Informed consent was practiced. The final sample size was based on data saturation, as defined by no new themes in the ongoing analysis of the interview data [36].

### 4.3 Phase 1. Qualitative Data Collection and Analysis Methods

The qualitative data collection and analysis was informed by Assaroudi et al.'s recommended methods for conducting a Directed Content Analysis [33]. Development of the semi‐structured interview was guided by insights from existing literature and the SEIPS model [27] by an ergonomist and a nurse. Individual interviews with the ICU nurse participants were conducted face‐to‐face by the first author in a private setting in the hospital. Each lasted around 60 min. The interviews were recorded and transcribed verbatim by two researchers and then imported into MAXQDA, a qualitative data analysis software used for organising, coding and analysing qualitative data. Coding rules were set by the research team. AI was not involved in the coding process in MAXQDA.

The deductive Direct Content Analysis was conducted simultaneously and continuously with data collection [33, 37]. Two researchers independently listened to the audio recordings and read through the transcripts on the same day to gain a general sense of the interview and become immersed in the data. The two researchers independently coded the interview transcripts line by line using an open, axial and selective coding approach. During the open coding phase, the data were meticulously examined to identify initial codes. On assurance of data saturation, the extracted codes were categorised based on similarities and differences. Axial coding was then applied to relate the codes to each other, forming coherent themes that represented potential interventions within the ICU work system. Finally, these themes were placed into one of the five components of the SEIPS model, according to their work system characteristics. The small number of coding differences was refined through discussion and consensus.

### 4.4 Phase 2. Data Integration for Weighting and Prioritisation Using Quantitative AHP

The identified potential interventions, grouped into the five SEIPS components, were weighted to enable prioritising future interventions using the Analytic Hierarchy Process (AHP)—a method for multi‐criteria decision‐making [38]. AHP involves transforming the qualitative data into numerical data by making pairwise comparisons of importance. Here, this involved the perceived workplace interventions to derive scales, which indicate priorities in relative (quantitative) terms [39, 40]. The process uses a scale of absolute judgements. This research used Saaty's scale, which has numerical values from 1 to 9, to make these comparisons [38].

The first step was to create a two‐level hierarchy. The first level comprised the five components of the SEIPS model (Person, Organisation, Technology and Tools, Tasks and Environment). For making pairwise comparison of the benefit of interventions at the component level, a 5 × 5 matrix was constructed. The second level comprised constructing five further matrices: one for each of the five components to compare the themes (representing interventions) associated with that component extracted from the interviews. Then, pairwise comparisons were conducted for all six matrices. The process of constructing comparison matrices is summarised below and explained more fully in the online Supporting Information (See Supporting Information—Developing a comparisons matrix).

Pairwise comparisons were made by the 14 ICU nurses (as expert participants) using an online AHP‐OS software tool [41]. This asked participants to compare all the potential interventions (derived from the qualitative themes) using pairwise comparisons according to Saaty's nine‐point scale [38]. That is, each participant was given a potential intervention as a reference, then asked to compare this reference intervention against another alternative intervention using the nine‐point scale to assign values. Thus, 1/9 is ‘extremely less important’ (the alternative is much more important); 1/7 means ‘very strongly less important’, 1/5: ‘strongly less important’, 1/3: ‘moderately less important’ and 1/1: ‘*equally important*’. (1/2, 1/4, 1/6, 1/8, serve as *‘*intermediate values between adjacent scale values’) [38]. This process was continued until all the potential interventions had been subject to a pairwise comparison with all other potential interventions. The AHP‐OS software then computed the consistency among the experts' comparisons, and local and global rankings were calculated.

### 4.5 Ethical Considerations

The research project investigating working conditions for ICU nurses was authorised by the Ethics Committee in Medical Research of Shiraz University of Medical Sciences under the code of ethics IR.SUMS.REC.1398.622 March 04, 2020. Additions for this research stream were approved March 01, 2023. All procedures followed the ethical standards of the responsible committee of human experimentation (institutional and national) and with the Helsinki Declaration of 1975, as revised in 2000. Written informed consent was obtained from all participants included in the study.

## 5 Results

Data saturation was achieved after the eleventh interview, as confirmed after 14 interviews. Thus the final sample included 14 ICU nurses, one of whom was the head nurse. Of these, four were male, and 10 were female. Five were ≤ 29 years, six aged 30–44 years, and three were ≥ 45. Ten had more than 5 years ICU experience, whilst the work experience of the other four was between one and 5 years.

### 5.1 Phase 1. Identified Interventions

**FIGURE 2:**
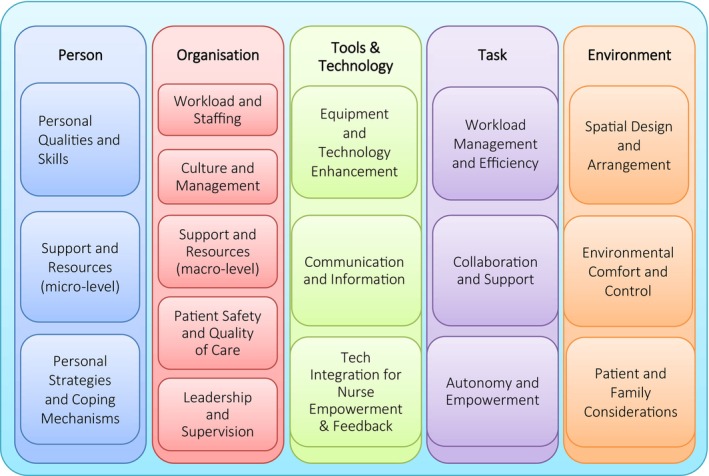
Identified themes aligned with the SEIPS model components.

Utilising the MAXQDA software, 348 raw codes were identified and thematically distributed across the five work system components of the SEIPS model. After axial and selective coding, 17 themes were extracted from the interviews (see Figure 2). These are described below, aligned with their SEIPS component and a few sample quotes, necessarily translated to English.

#### 5.1.1 Person

The Person component focuses on individual attributes of nurses, micro‐level support systems (e.g., emotional support, financial support, verbal appreciation from management) and personal stress management techniques. Under this component, three themes were identified:

1. Personal Qualities and Skills. Participants believed that nurses in the ICU must learn some characteristic features such as having a positive attitude, being willing to adapt and possession of self‐awareness and emotional intelligence.
*As nurses, we must improve our knowledge. When a nurse comes to this department, they limit themselves to following a series of instructions. Of course, we have defined tasks anyway, I can't do surgery on the patient, but I can get more information about their medications, gain updated knowledge about their post‐operative care and improve myself in these things*.
2. Support and Resources (micro‐level). Participants mentioned the essential need for supportive and complementary programmes including ongoing training and development and gaining management support and recognition.
*My suggestion is that a nurse who comes to the ICU department must undergo a series of trainings, especially for nurses who are just starting to work*.
3. Personal Strategies and Coping Mechanisms. Given the demanding nature of the ICU department, where patients are often admitted in critical condition, nurses consistently emphasised that individuals working in this environment must gain the ability to maintain a healthy work‐life balance, engage in self‐care and stress management practices, and build resilience and adaptability.

*If a happy mood and atmosphere is created, it can be useful*.

#### 5.1.2 Organisation

Five themes emerged as influencing operational environments and patient care quality: Workload and Staffing, Culture and Management, Support and Resources (macro‐level), Patient Safety and Quality of Care, and Leadership and Supervision.

1. Workload and staffing related to having balanced workloads, appropriate staffing levels and flexible scheduling practices as the core ideas for improving the work system for the ICU nurses.

2 Culture and Management. ICU nurses identified several cultural and management concepts as substantial fields through which their work environment can be enhanced. These encompassed a positive and supportive work environment, a collaborative management style, respectful communications and recognition of nurses' efforts, all of which can foster a sense of belonging.
*If a mistake happens, how a supervisor reacts and gives feedback can really help the nurse learn from it, instead of getting defensive because the criticism was given the wrong way*.
3 Support and Resources (macro‐level). In this area of organisational strategies, effective leadership and supervision were the main code. Competent and supportive leaders, who provide guidance and feedback, create a positive work environment and promote nurses' development.
*For example, here, for CPR, they can send some nurses from this department to hospitals in bigger cities, so that they can learn it, and then they can teach it to others back here*.
4 Patient Safety and Quality of Care. Provision of all the necessary equipment, technology and training opportunities enables nurses to perform their duties effectively and remain competent.
5 Leadership and Supervision. Organisations can put more emphasis on creating a work environment that fosters nurses' well‐being and enables them to provide high‐quality care to patients. Points included increased cooperation between supervisor and nurses, as this motivates nurses and contributes to their professional fulfillment.

*In my opinion, it would be much better if the ICU had a resident doctor*.

*Look, there should be a better balance between the number of patients and nurses so that we can give better care to the patient and manage our time*.

*In my opinion, it would be much better if the ICU had a resident doctor*.

*Look, there should be a better balance between the number of patients and nurses so that we can give better care to the patient and manage our time*.

#### 5.1.3 Technology and Tools

In this component, Equipment and Technology Enhancement, Communication and Information, Management Improvement and Tech Integration for Nurse Empowerment and Feedback were areas for intervention. Nurses highlighted the need for regular checks and renewal of dated tools, improved communication systems and the latest technology to empower nurses.

1. Equipment and Technology Enhancement. Nurses emphasised how regular checks on equipment were needed. They explained that providing adequate training and access to essential equipment facilitates safe and efficient patient care whilst reducing nurse mental workload. Moreover, implementing features like patient bathrooms, minimising physical restraints and adjusting bed heights improved the patient care process and reduced nurse burden.
2. Communication and Information Management Improvement. Utilising effective communication and information sharing technologies streamlines workflow, optimises decision‐making and fosters collaboration, reducing current times of nurse frustration and promoting teamwork.
3. Technology Integration for Nurse Empowerment & Feedback. Nurses indicated that incorporating opportunities for them to provide work‐related suggestions and participate in equipment decisions strengthens their sense of autonomy and respect. All the nurses agreed that this would promote their motivation.

#### 5.1.4 Tasks

Themes included Workload Management and Efficiency, Collaboration and Support, and Autonomy and Empowerment, stressing effective workload management, collaborative work environments and empowering nurses.

1. Workload Management and Efficiency. Nurses suggested strategies to reduce their workload, enhance prioritisation of tasks according to circumstance and generally improve efficiency.
2. Collaboration and Support. Fostering teamwork, clear communication and interprofessional collaboration were the main interventions that our participants believed can enhance workflow, reduce conflicts and create a more supportive environment.
3. Autonomy and Empowerment. Providing autonomy, recognising expertise and fostering professional identity were referred to as the basic concepts under this theme.

#### 5.1.5 Environment

In the Environment component, Spatial Design and Arrangement, Environmental Comfort and Control, and Patient and Family Considerations underscored the importance of conducive physical spaces, comfort and considerations for patients and their families.

### 5.2 Phase 2. Prioritisation of Identified Interventions

The results of the participants' pairwise comparisons of the interventions grouped by SEIPS components are presented in Table 1. Organisation was identified as the most crucial component requiring intervention for optimisation. Person was ranked as second in importance, then Tasks, Environment and Tools and Technology.

**TABLE 1:** AHP prioritisation of the SEIPS work system components.

Matrix	Person	Organisation	Tools & technology	Tasks	Environment	Weight	Rank
Person	1	0.59	1.33	1.44	1.20	0.206	2
Organisation	1.68	1	1.98	1.60	1.98	0.309	1
Tools & technology	0.75	0.50	1	0.79	0.96	0.150	5
Tasks	0.70	0.62	1.27	1	1.31	0.181	3
Environment	0.83	0.50	1.04	0.76	1	0.154	4

*Note:* CR: 0.4%, CI: 0.02, AHP group consensus: 83.6%.

The weights and rankings that emerged from the pairwise comparisons of the interventions at the SEIPS component level are summarised in Table 2. (The outcomes of the five comparison matrices are laid out in full in Tables S1, S2, S3, S4 and S5 in the online Supporting Information.) According to the 14 ICU nurses in this study, for Person, Support and Resources was ranked as most important for future interventions, and for Organisation, most important was Workload and Staffing. Regarding Technology and Tools, Equipment and Technology Enhancement was the most important, and for the Tasks interventions, ICU nurses emphasised the importance of Collaboration and Support. In the Environment component, intervention to improve Spatial Design and Arrangement was weighted as the highest priority.

**TABLE 2:** Weights and rankings of potential interventions based on the SEIPS model.

SEIPS component	Relative weight	Themes	Local weight	Local rank	Global weight	Global normalised	Global rank
Person	0.206	Personal qualities and skills	0.27	2	0.056	5.56%	7
		Support and resources (micro‐level)	0.542	1	0.112	11.17%	2
		Personal strategies and coping mechanisms	0.188	3	0.039	3.87%	13
Organisation	0.309	Workload and staffing	0.374	1	0.116	11.56%	1
		Culture and management	0.248	2	0.077	7.66%	5
		Support and resources (macro‐level)	0.117	4	0.036	3.62%	15
		Patient safety and quality of care	0.175	3	0.054	5.41%	8
		Leadership and supervision	0.086	5	0.027	2.66%	16
Tools & technology	0.15	Equipment and technology enhancement	0.570	1	0.085	8.54%	4
		Communication and information	0.163	3	0.024	2.44%	17
		Tech integration for nurse empowerment & feedback	0.268	2	0.040	4.02%	11
Tasks	0.181	Workload management and efficiency	0.257	2	0.047	4.65%	9
		Collaboration and support	0.538	1	0.097	9.73%	3
		Autonomy and empowerment	0.205	3	0.037	3.72%	14
Environment	0.154	Spatial design and arrangement	0.459	1	0.071	7.06%	6
		Environmental comfort and control	0.251	3	0.039	3.87%	12
		Patient and family considerations	0.29	2	0.045	4.47%	10

The global weights of the preferred interventions across the five work system components were evaluated for their overall importance in the study. The highest prioritised were Workload and Staffing, and Support and Resources (micro‐level). Notable global weights were also indicated for Collaboration and Support, Equipment and Technology Enhancement, Culture and Management, and Spatial Design and Arrangement. Interventions to improve Patient Safety and Quality of Care, Workload Management and Efficiency, and Patient and Family Considerations were rated less important, whilst Technology Integration for Nurse Empowerment and Feedback, Autonomy and Empowerment, Environmental Comfort and Control, and Personal Strategies and Coping Mechanisms had lower emphasis. The AHP indicated that Support and Resources (macro‐level), Leadership and Supervision, and Communication and Information were considered less critical.

## 6 Discussion

The aim of this study was to identify and prioritise interventions to support improving the work system in ICU by gathering input from a sample of experienced critical care nurses. Analysis of data from fourteen searching interviews found 17 themes associated with ICU intervention, which could be aligned with the five components of the established SEIPS model [27]. The subsequent use of AHP [38] to rank the ICU nurses' comparisons of importance of the 17 potential interventions highlighted that Organisation includes a key component in improving the ICU work system. The participants also reported potentials for intervention in the other four components that would improve well‐being and job performance for ICU nurses, particularly Person, then Tasks, Environment and Technology and Tools. Of the 17 identified interventions, the most significant were Workload and Staffing, followed by Support and Resources (micro‐level), Collaboration and Support, and Equipment and Technology Enhancement.

The finding that the Organisation and Person components play a crucial role in the work system, being responsible for more than half of the global weights, aligns with a systematic review of studies investigating barriers to patient and family‐centred care in adult ICU [42], which identified four types of barriers to patient and family‐centred care: lack of understanding, organisational barriers, individual barriers and interdisciplinary barriers. These barriers can also compound each other, as highlighted by a systematic review of staff‐reported barriers and facilitators in the implementation process of general hospital interventions [43]. One major limitation mentioned in the latter article, however, was that most studies collected qualitative data but lacked sufficient details regarding the interview and data analysis methods used, indicating methodological shortcomings in the field. Nevertheless, the authors concluded that for researchers and health professionals involved in designing patient‐focused interventions, it is crucial to consider barriers and facilitators across all work system components to enhance the chances of successful implementation of interventions [43].

According to the ICU nurses in this study, the area most in need of improvement is Workload and Staffing. A range of workplace interventions associated with this theme, including flexible work arrangements, job modifications and participatory process initiatives, have previously been found to improve the well‐being of healthcare employees, particularly in the areas of job satisfaction, work‐family balance and mental health generally [44]. Appropriate interventions can also reduce burnout, improve work ability and enhance overall health and well‐being in healthcare settings [45, 46]. Altogether, this was a significant reason to seek out perceived priorities for interventions to improve ICU nurses' well‐being, which is a vital precursor to high‐quality patient care [46].

The significance of interpersonal behaviours in creating a supportive work environment was also evident in priorities for interventions. Support and Resources at the micro‐level range from tangible financial compensation and assistance from peers to intangible resources like verbal appreciation and emotional support from management. Exploring the specific types of support lacking among team members and implementing practical solutions could enhance the overall work atmosphere. Furthermore, the nurse interviews confirmed that emphasising interpersonal behaviours such as verbal appreciation and emotional support is good practice. They underscore the importance of establishing a psychologically safe and positive workplace, and a leader's emotional management significantly impacts the team's well‐being and overall work climate [47]. There is also evidence that providing constructive feedback that is positive and specific is associated with higher levels of employee engagement and performance compared to negative or vague feedback [48]. This supports the notion of giving private, error‐specific feedback instead of public criticism, in line with the qualitative findings from this research. However, studies on positive and negative feedback, frequency and feedback properties in the workplace are still limited and require further exploration. Additionally, the results of this meta‐analysis study [48] were based on self‐reported measures, which may be considered less reliable than the mixed‐methods research design used in this study, accepting that participant numbers were small for the quantitative phase. Altogether, the outcomes raise intriguing possibilities for introducing leadership training in emotional regulation to cultivate a culture of mutual support among all team members to foster a more harmonious work environment. Recognising the interconnected nature of support, resources and interpersonal dynamics is essential in developing comprehensive strategies for organisational enhancement.

Another feasible intervention that came out of the current study was to provide greater collaboration and task support. This would include collaboration and delegation, peer support and transparency and openness. The importance of implementing interventions like teamwork and leadership training to enhance patient safety outcomes in an emergency context has been raised before [48]. Conclusions from that study were that implementing a combination of intervention methods for teamwork improvement, particularly focusing on resource management training related to teamwork, communication, workload management, stress and fatigue management, leadership, decision‐making and handling adverse situations, can lead to significant cultural changes [49]. Additionally, enhanced communication skills among colleagues may extend to interactions with patients and their families [50].

In our research, participants also emphasised that equipment, technology, workplace environment and facilities are crucial for ensuring both patient safety and their own welfare. Other studies have similarly identified insufficient support and resources as barriers to these goals [23, 29, 31, 42]. Further, a systematic review concluded that implementing a range of interventions related to support and resources in the workplace, such as physical activity facilities and dietary interventions, does lead to a significant reduction in sickness absence levels and a significant uplift in job satisfaction and physical activity levels [51].

### 6.1 Limitations

The research was conducted with a sample of fourteen ICU nurses employed in a single tertiary care hospital. Whilst data saturation was assured, it remains that their experience may not adequately represent the experiences and opinions of nurses in other ICU. Additionally, although the qualitative data collection phase attempted to take a longitudinal perspective, it can only provide a snapshot of the opinions and experiences in an ICU work system at one point in time. Thus, the interventions identified in this study may not capture the dynamic challenges faced over time, or in different institutional settings, with different work environments, or in different cultures with respect to the social value of nursing [52] in this ever evolving field of healthcare. It was important to use the same fourteen participants in the two phases of the study. We would recommend, however, that future studies that use this methodology supplement the sample with additional participants in the phase 2 quantitative pairwise comparisons that support the AHP used to weight and rank the preferred interventions to support the decision‐making process involved in planning interventions with limited resources. This would also ascertain whether the lower rankings for themes such as Patient Safety and Quality of Care, Workload Management and Efficiency, and Patient and Family Considerations were rated less important in this study because these aspects of the work system are currently being managed well in the ICU in this setting, and whether more attention to this ‘core business’ of ICU would be beneficial in other ICU settings. Ultimately, future studies with larger and more diverse samples across multiple hospitals and including a range of healthcare professionals could expand on these findings and offer more comprehensive recommendations for practice and policy to those presented as feasible in this study.

### 6.2 Implications for Practice

Our research, in line with previous studies, suggests a need for immediate and long‐term solutions to address the demanding workloads and staffing challenges faced by critical care nurses. Whilst based on a limited cohort of ICU nurses in one hospital, the robust methodology and theoretical underpinning indicate that interventions that focus on organisational aspects like workload and staffing, as well as individual needs for support and resources towards introducing meaningful changes could improve nurse well‐being and patient care in critical care settings. This requires practical attention being regularly made to ensuring suitable and sufficient nurse–patient ratios for both nurse well‐being and high‐quality patient care [46]. The nurses in this study indicated a need for immediate actions that included revisiting extant staffing policies alongside making systems and technological changes; implementing mental health support programmes to promote resilience in this inherently stressful setting; and enhancing professional development opportunities for ICU nurses to keep up with technological improvements as well as career enhancement. Supporting the continuous education of ICU nurses equips them with the skills necessary to handle evolving healthcare technologies and practices [33]. Similarly, investment in advanced healthcare technology and implementation of mental health programmes are vital to reduce workloads, improve efficiency and create a more supportive work environment. Fostering a collaborative work environment and strengthening policies for interprofessional collaboration will promote communication and teamwork across healthcare disciplines, ultimately leading to better patient outcomes.

## 7 Conclusion

The sequential exploratory mixed‐methods study used the SEIPS model as a framework to identify and then rank 17 potential interventions that could improve work systems in ICU for nurses. The research advances the literature in terms of providing a robust methodology for prioritising evident requirements for interventions. The findings lay the groundwork for driving forward improvements in ICU work systems, nurse well‐being and performance and ultimately patient care. By addressing the multifaceted challenges of ICU environments through tailored and comprehensive interventions, this research offers a valuable approach for understanding and meeting the complex needs of ICU nurses and ultimately enhancing the overall functionality of critical care work systems.

## Funding

There was no external financial support associated with this research. This research was supported by Shiraz University of Medical Sciences in terms of resources (Grant number 17001). This support did not influence the activity associated with the research in any way.

## Ethics Statement

The research project investigating working conditions for ICU nurses was authorised by the Ethics Committee in Medical Research of Shiraz University of Medical Sciences under the code of ethics IR.SUMS.REC.1398.622 March 04, 2020. Additions for this research stream were approved March 01, 2023. All procedures followed the ethical standards of the responsible committee of human experimentation (institutional and national) and with the Helsinki Declaration of 1975, as revised in 2000. Written informed consent was obtained from all participants included in the study.

## Conflicts of Interest

The authors declare no conflicts of interest.

## Supporting information


**Data S1:** Supporting Information—Developing a comparisons matrix.

**Table S1:** SEIPS Component Matrix: Person.

**Table S2:** SEIPS Component Matrix: Organisation.

**Table S3:** SEIPS Component Matrix: Tools and Technology.

**Table S4:** SEIPS Component Matrix: Task.

**Table S5:** SEIPS Component Matrix: Environment.

## Data Availability

The data generated and analysed during this research are not publicly available. Data sharing was not included in the ethical approval as the study includes personal accounts of participants' working conditions, which could be sensitive. It is essential we keep confidentiality and anonymity; however, sufficiently anonymised parts of the data are available for the corresponding authors upon reasonable request.
