# Supplementary material for: Preferences of ICU Nurses for Improving Their Work System: A Sequential Exploratory Mixed‐Methods Study
Source: Nurs Crit Care. 2026 Feb 2;31(2):e70350. doi: 10.1111/nicc.70350 (PMC12863987; doi:10.1111/nicc.70350)
Supplement: Supplementary file 1 — Data S1: Supporting Information—Developing a comparisons matrix. [file NICC-31-0-s001.docx]

Supplementary Information

**Developing a comparisons matrix**

The process of constructing a comparison matrix in the AHP model, specifically when evaluating alternatives against a given criterion is presented here:

**Structure:**

- **n x n Matrix**: The matrix A is a square matrix where n represents the number of alternatives being evaluated. Each row and column correspond to one of the alternatives.

**Values:**

- ***a_ij_*_​_**: Each element in the matrix, denoted as *a_ij_*_​_, represents the relative importance of alternative *i* compared to alternative *j* concerning a specific criterion.
  - **Row *i***: This indicates the alternative being evaluated.
  - **Column *j***: This indicates the alternative being compared against.

**Importance Scale:**

- **Strong Importance**: If alternative *i* is considered significantly more important than alternative *j* with respect to the criterion, the value assigned to *a_ij_*_​_is 5. This indicates a strong dominance of *i* over *j*.
- **Intermediate Values**: The method allows for intermediate values to be used, which represent varying degrees of importance. For example, if the importance of *i* over *j* is moderate, it might receive a value of 3.

**Matrix Relationships:**

- The construction of the matrix relies on certain relationships:
  - **Reciprocal Relationships**: If *a_ij_*_​_is the value for the comparison of *i* over *j*, then *a_ji_*​ (the comparison of *j* over *i*) will be the reciprocal of *a_ij_*_​_. For example, if *aij*​=5, then *aji*​=1/5​.

| *a_ij_*_​ =_ $\frac{1}{a_{ij}}$ | Eq.1 |
| --- | --- |

for consistent evaluations:

| $a_{jk}=a_{ik}/a_{ij}$ | Eq.2 |
| --- | --- |

where $\boldsymbol{k}$ and $\boldsymbol{j}$ are two alternatives being compared to $\boldsymbol{i}$.

After completing the matrix, the process finds a priority vector for each alternative based on the criterion. According to Saaty, this vector is derived from the relationship between matrix A and its largest eigenvalue.

We can calculate the consistency index $(CI)$ with this formula:

$CI=(\lambda max-n)/(n-1)$ Eq.3

$$\lambda max:Maximum eigenvalue of the pairwise comparison matrix$$

n: the number of criteria

Count consistency ratio (CR) with this formula:

$CR=CI/RI$ Eq.4

where: $CR=$ Consistency Ratio, $RI=$ Random Consistency Index (varies based on the size of the matrix.

In AHP, the consistency ratio (CR) should be ≤10% for reliable results. CR measures the consistency of responses. CR>10% means judgments are untrustworthy, while CR≤10% means consistent judgments [38]. Also, The AHP group consensus rate is an indicator that assesses the extent of alignment in priority setting among group members. It serves as a gauge for the level of “agreement” within the group, with a spectrum that spans from 0% (indicating no consensus) to 100% (indicating complete consensus). This spectrum is divided into five classifications: very low, low, moderate, high, and very high. The AHP group consensus rates were acquired using the AHP Survey Tool [41].
