# Supplementary material for: Preferences of ICU Nurses for Improving Their Work System: A Sequential Exploratory Mixed‐Methods Study
Source: Nurs Crit Care. 2026 Feb 2;31(2):e70350. doi: 10.1111/nicc.70350 (PMC12863987; doi:10.1111/nicc.70350)
Supplement: Supplementary file 2 — Table S1: SEIPS Component Matrix: Person. [file NICC-31-0-s004.docx]

Table S1. SEIPS Component Matrix: Person

| Matrix | Personal Qualities and Skills | Support and Resources (micro-level) | Personal Strategies and Coping Mechanisms | Weight | Rank |
| --- | --- | --- | --- | --- | --- |
| Personal Qualities and Skills | 1 | 0.48 | 1.50 | 0.270 | 2 |
| Support and Resources (micro-level) | 2.10 | 1 | 2.76 | 0.542 | 1 |
| Personal Strategies and Coping Mechanisms | 0.67 | 0.36 | 1 | 0.188 | 3 |
| CR: 0.2%, CI: 0.01, AHP group consensus: 82.3% | | | | | |
