# Supplementary material for: Preferences of ICU Nurses for Improving Their Work System: A Sequential Exploratory Mixed‐Methods Study
Source: Nurs Crit Care. 2026 Feb 2;31(2):e70350. doi: 10.1111/nicc.70350 (PMC12863987; doi:10.1111/nicc.70350)
Supplement: Supplementary file 3 — Table S2: SEIPS Component Matrix: Organisation. [file NICC-31-0-s005.docx]

Table S2. SEIPS Component Matrix: Organization

| Matrix | Workload and Staffing | | Culture and Management | Support and Resources (macro-level) | Patient Safety and Quality of Care | Leadership and Supervision | Weight | Rank |
| --- | --- | --- | --- | --- | --- | --- | --- | --- |
| Workload and Staffing | | 1 | 1.6 | 2.9 | 3.0 | 3.0 | 0.374 | 1 |
| Culture and Management | | 0.61 | 1 | 2.47 | 1.53 | 2.68 | 0.248 | 2 |
| Support and Resources (macro-level) | | 0.34 | 0.40 | 1 | 0.54 | 1.79 | 0.117 | 4 |
| Patient Safety and Quality of Care | | 0.33 | 0.65 | 1.86 | 1 | 2.49 | 0.175 | 3 |
| Leadership and Supervision | | 0.33 | 0.37 | 0.56 | 0.40 | 1 | 0.086 | 5 |
| CR: 2.1%, CI: 0.08, AHP group consensus: 95% | | | | | | | | |
