# Supplementary material for: Preferences of ICU Nurses for Improving Their Work System: A Sequential Exploratory Mixed‐Methods Study
Source: Nurs Crit Care. 2026 Feb 2;31(2):e70350. doi: 10.1111/nicc.70350 (PMC12863987; doi:10.1111/nicc.70350)
Supplement: Supplementary file 4 — Table S3: SEIPS Component Matrix: Tools and Technology. [file NICC-31-0-s006.docx]

Table S3. SEIPS Component Matrix: Tools and Technology

| Matrix | Equipment and Technology Enhancement | Communication and Information | Tech Integration for Nurse Empowerment & Feedback | Weight | Rank |
| --- | --- | --- | --- | --- | --- |
| Equipment and Technology Enhancement | 1 | 3.15 | 2.36 | 0.57 | 1 |
| Communication and Information | 0.32 | 1 | 0.55 | 0.16 | 3 |
| Tech Integration for Nurse Empowerment & Feedback | 0.42 | 1.83 | 1 | 0.27 | 2 |
| CR: 1.1%, CI: 0.03, AHP group consensus: 93% | | | | | |
