# Supplementary material for: Preferences of ICU Nurses for Improving Their Work System: A Sequential Exploratory Mixed‐Methods Study
Source: Nurs Crit Care. 2026 Feb 2;31(2):e70350. doi: 10.1111/nicc.70350 (PMC12863987; doi:10.1111/nicc.70350)
Supplement: Supplementary file 5 — Table S4: SEIPS Component Matrix: Task. [file NICC-31-0-s002.docx]

Table S4. SEIPS Component Matrix: Task

| Matrix | Workload Management and Efficiency | Collaboration and Support | Autonomy and Empowerment | Weight | Rank |
| --- | --- | --- | --- | --- | --- |
| Workload Management and Efficiency | 1 | 0.43 | 1.38 | 0.26 | 2 |
| Collaboration and Support | 2.31 | 1 | 2.37 | 0.54 | 1 |
| Autonomy and Empowerment | 0.72 | 0.42 | 1 | 0.20 | 3 |
| CR: 1.1%, CI:0.03, AHP group consensus: 94% | | | | | |
