# Supplementary material for: Preferences of ICU Nurses for Improving Their Work System: A Sequential Exploratory Mixed‐Methods Study
Source: Nurs Crit Care. 2026 Feb 2;31(2):e70350. doi: 10.1111/nicc.70350 (PMC12863987; doi:10.1111/nicc.70350)
Supplement: Supplementary file 6 — Table S5: SEIPS Component Matrix: Environment. [file NICC-31-0-s003.docx]

Table S5. SEIPS Component Matrix: Environment

| Matrix | Spatial Design and Arrangement | Environmental Comfort and Control | Patient and Family Considerations | Weight | Rank |
| --- | --- | --- | --- | --- | --- |
| Spatial Design and Arrangement | 1 | 1.77 | 1.63 | 0.46 | 1 |
| Environmental Comfort and Control | 0.57 | 1 | 0.84 | 0.25 | 3 |
| Patient and Family Considerations | 0.61 | 1.19 | 1 | 0.29 | 2 |
| CR: 0.1%, CI:0.001, AHP group consensus: 84% | | | | |  |
